# Intensive consolidation therapy compared with standard consolidation and maintenance therapy for adults with acute myeloid leukaemia aged between 46 and 60 years: final results of the randomized phase III study (AML 8B) of the European Organization for Research and Treatment of Cancer (EORTC) and the Gruppo Italiano Malattie Ematologiche Maligne dell’Adulto (GIMEMA) Leukemia Cooperative Groups

**DOI:** 10.1007/s00277-012-1436-z

**Published:** 2012-03-31

**Authors:** Marysia Hengeveld, Stefan Suciu, Matthias Karrasch, Giorgina Specchia, Jean-Pierre Marie, Petra Muus, Maria C. Petti, Bruno Rotoli, Sergio Amadori, Guiseppe Fioritoni, Pietro Leoni, Enrica Morra, Joseph Thaler, Luigi Resegotti, Paola Fazi, Marco Vignetti, Franco Mandelli, Robert Zittoun, Theo de Witte

**Affiliations:** 1 Radboud University Nijmegen Medical Center, Nijmegen, The Netherlands; 2 EORTC Headquarters, Brussels, Belgium; 3 Policlinico Bari, Bari, Italy; 4 Hôtel-Dieu Paris, Paris, France; 5 Sapienza, Rome, Italy; 6 Sc Med. Univ Napoli, Naples, Italy; 7 Ospedale San Eugenio, Rome, Italy; 8 Ospedale Civile Pescara, Pescara, Italy; 9 Ospedale Riuniti di Ancona, Ancona, Italy; 10 Ospedale Niguarda Ca’Granda, Milan, Italy; 11 Medizinische Universitätsklinik, Innsbruck, Austria; 12 Le Molinette, Turin, Italy; 13 GIMEMA Data Center, Rome, Italy

**Keywords:** Acute myeloid leukaemia, Post-remission chemotherapy

## Abstract

The most effective post-remission treatment to maintain complete remission (CR) in adults aged between 46 and 60 years with acute myeloid leukaemia (AML) is uncertain. Previously untreated patients with AML in CR after induction chemotherapy with daunorubicin and cytarabine were randomized between two intensive courses of consolidation therapy containing high-dose cytarabine, combined with amsacrine or daunorubicin and a standard consolidation and maintenance therapy containing standard dose cytarabine and daunorubicin. One hundred fifty-eight CR patients were assigned to the intensive group and 157 patients to the standard group. After a median follow-up of 7.5 years, the 4-year survival rate was 32 % in the intensive group versus 34 % in the standard group (*P* = 0.29). In the intensive group, the 4-year relapse incidence was lower than in the standard group: 55 and 75 %, respectively (*P* = 0.0003), whereas treatment-related mortality incidence was higher: 22 versus 3 % (*P* < 0.0001). Two intensive consolidation courses containing high-dose cytarabine as post-remission treatment in patients with AML aged between 46 and 60 years old did not translate in better long-term outcome despite a 20 % lower relapse incidence. Better supportive care and prevention of treatment-related complications may improve the overall survival after intensified post-remission therapy in this age group.

## Introduction

Patients, younger than age 60 years, with acute myeloid leukaemia (AML) may achieve complete remission (CR) in 60 to 80 % of the cases treated with intensive remission induction courses usually consisting cytarabine (Ara-C) combined with an anthracycline/anthracycline-like drug [1–5]. Following remission induction therapy, additional treatment is important, as the median relapse-free survival (RFS) for patients who do not receive additional therapy is only 4–8 months [4]. Therefore, most patients receive post-remission therapy, which may consist of consolidation/maintenance chemotherapy, autologous or allogeneic stem cell transplantation [2–4]. Both allogeneic and autologous stem cell transplantation is associated with higher treatment-related morbidity and mortality patients with the age between 46 and 60 years compared to younger patients [6]. Therefore these patients are usually treated with consolidation and/or maintenance chemotherapy only [2, 4]. Despite many studies on post-remission therapy to prevent relapse with chemotherapy with maximal efficiency and minimal toxicity, an effective schedule for this age group has not been established until now [2, 4, 7–15].

Cytarabine plays a central role in the post-remission treatment of AML, but exact doses and number of cycles have not been defined yet [2, 4]. Cytarabine has a steep dose–response curve, which stimulated many study groups to verify this effect in prospective trials on post-remission treatment of AML [2, 8, 10, 14–21]. The European Organization for Research and Treatment of Cancer (EORTC) and the Gruppo Italiano Malattie Ematologiche Maligne dell’Adulto (GIMEMA) Leukemia Cooperative Groups developed a prospective randomized phase III trial in patients with AML aged between 46 and 60 years, to test two different dosages of cytarabine in post-remission treatment. All patients received induction therapy consisting of a combination of cytarabine and daunorubicin after which they were randomly assigned to receive two courses of intensive consolidation therapy containing high-dose cytarabine, combined with either amsacrine or daunorubicin or to receive a standard consolidation and maintenance course containing standard dose cytarabine and daunorubicin. The group performed simultaneously a study in patients younger than 45 years on the role of allogeneic and autologous stem cell transplantation after intensive consolidation chemotherapy alone [22].

## Methods

### Patients

Patients with previously untreated acute myeloid leukaemia were eligible for entry into the trial. The diagnosis was made by the participating centres according to the criteria of the French–American–British (FAB) classification system [23]. A cytology committee reviewed the smears from 53 % of patients. Patients with age between 46 and 60 years were eligible, but some centres which did not perform stem cell transplantation were allowed to enrol younger patients. Informed consent was obtained according to the regulations of each institution. Patients with chronic myeloid leukaemia or other myeloproliferative diseases in blast crisis were excluded, as were patients who had leukaemia supervening after other myeloproliferative diseases or patients who had a preceding myelodysplastic syndrome for more than 6 months. Most centres excluded patients with acute promyelocytic (M3) leukaemia. Patients with concomitant hepatic, renal, cardiac or neurological disease or patients with other progressive malignant diseases have been excluded as well.

### Study design

The induction treatment consisted of one course, or in case of partial remission, two courses, consisting of daunorubicin, at a dose of 45 mg/m^2^ of body surface area, given intravenously as a push infusion on days 1, 2 and 3 and cytarabine at a daily dose of 200 mg/m^2^ given as a continuous intravenous infusion on days 1 through 7. In case of leukemic regrowth, absolute resistance or persistent hypoplasia, a salvage scheme was used, which consisted of idarubicin, at a dose of 12 mg/m^2^ of body surface area given as a continuous infusion on days 1, 3 and 5, and cytarabine at a dose of 500 mg/m^2^ given as a continuous intravenous infusion over a period of 2 h every 12 h on days 1 through 7 [24].

**Fig. 1:**
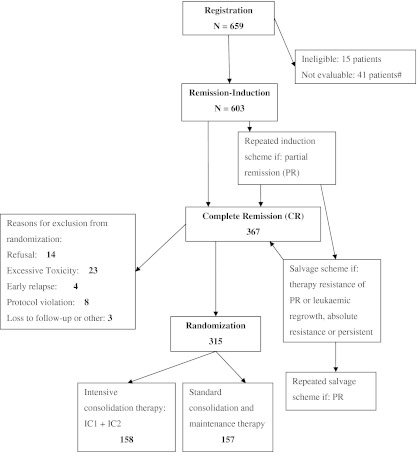
Design of the study. The *numbers* are the numbers of patients at each treatment step. Details of the treatment are given in the “Methods” section. The 659 registered patients included 37 patients who participated to the GM-CSF study [25]. Seventeen patients have been randomized, but they have not been included in this study. Of 603 patients, 367 reached CR, 157 patients were excluded because they did not reach CR and 79 patients died before they could reach CR. *IC1* first course of intensive treatment schedule. *IC2* second course of intensive treatment schedule

All patients who achieved complete remission were randomized between short intensive consolidation therapy or standard consolidation and maintenance therapy, except 17 patients who participated to an amendment of the trial to study the role of GM-CSF during remission induction therapy [25]. For details, see Fig. 1.

Short intensive consolidation treatment consisted of two courses. The first course contained cytarabine, at a dose of 500 mg/m^2^ of body surface given as a continuous intravenous infusion over a period of 2 h every 12 h on days 1 through 6, and amsacrine at a dose of 120 mg/m^2^, given in a 3-h intravenous infusion on days 5, 6 and 7. The second course contained cytarabine at a dose of 2 g/m^2^ given in an intravenous infusion over a period of 2 h every 12 h on days 1 through 4 and daunorubicin at a dose of 45 mg/m^2^ given as a push infusion on days 5, 6 and 7. The dose of cytarabine was limited to 2 g/m^2^ to decrease the risk of cerebellar toxicity.

Standard consolidation maintenance treatment consisted of two parts. The consolidation treatment consisted of one course with cytarabine at a dose of 200 mg/m^2^ given as a continuous infusion on days 1 through 7 and daunorubicin at a dose of 45 mg/m^2^ given as a push infusion on day 1. The maintenance treatment consisted of six courses with daunorubicin at a dose of 45 mg/m^2^ given as a push intravenous infusion on day 1 and cytarabine at a dose of 100 mg/m^2^ given subcutaneously every 12 h on days 1 through 5, given at 4- to 6-week intervals.

The Italian centres used a similar standard consolidation/maintenance treatment which consisted of four courses which contained daunorubicin at a dose of 60 mg/m^2^, given intravenously on day 1; cytarabine at a dose of 60 mg/m^2^, given subcutaneously every 8 h on days 1 till 5 and thioguanine at a dose of 70 mg/m^2^ of body surface area, orally every 8 h on days 1 till 5. Sequential courses were given with 4-week intervals. Several Italian centres without transplant facilities elected to include patients younger 46 years.

### Criteria for evaluation

A complete remission (CR) was defined according to the criteria of the CALGB [26]. Both bone marrow and peripheral blood should remain normal for at least 1 month. Leukemic cell infiltrations of the skin or of other sites should not be present. A partial remission (PR) was defined as a reduction of marrow blasts of at least 50 and 5.1–25 % marrow blasts with <5 % circulating blasts. A complete remission lasting less than 1 month was classified as a PR. Relapse was defined as more than 10 % leukemic cells in bone marrow aspirates or new extramedullary leukaemia in patients with a previously documented CR. Extramedullary relapse should be defined by cytology or histology.

### Statistical considerations

All patients were prospectively registered at the EORTC Data Centre in Brussels, and those considered to fulfil eligibility criteria for randomization after the achievement of complete remission were allocated to receive either standard consolidation and maintenance therapy (control arm) or short intensive consolidation therapy (experimental arm). The primary objectives of the study were to assess the value of short intensive consolidation chemotherapy versus a standard maintenance therapy in terms of RFS and overall survival (OS). The secondary objectives were to assess toxicities of these two types of post-remission therapy. A total of 300 patients had to be randomized in order to observe 182 events (relapses or deaths) which would allow to detect an increase from 25 to 40 % of patients alive and without relapse at 3 years, corresponding to a hazard ratio (HR; ratio of the instantaneous event rate in the experimental group versus the control group) of 0.66. RFS was calculated from the date of randomization until the date of the first relapse or the date of death in first complete remission. The OS was defined as the length of time from the date of randomization to the date of death. Survival distributions were estimated according to the Kaplan–Meier technique, and the standard errors (SE) of the estimates were obtained using Greenwood’s formula [27]. The differences between curves were tested for statistical significance with the two-tailed log-rank test [27]. The Cox’s proportional hazards model was used to obtain the estimate and the 95 % confidence interval (CI) of the HR [27]. Competing risk methods were used to estimate the incidences of relapse and of death in CR along with their SEs, and to perform their comparison (the Gray test) [27]. All randomized patients were analysed in their respective treatment groups in order to adhere to the intention-to-treat principle.

## Results

Between November 1986 and April 1993, 659 patients have been registered in the study by 44 institutions. Figure 1 shows the flow chart of the study. Fifteen patients were ineligible (6 patients because of chronic myeloid leukaemia, 1 patient because of blast crisis, 1 because of myelodysplastic syndrome with a duration longer than 6 months, 4 because of other diseases which met the exclusion criteria, 1 because the age did not fulfil the inclusion criteria and 2 because of other malignancies), and 41 patients could not be evaluated, including 37 patients who participated to the GM-CSF study [25]. Thus, a total of 603 patients were evaluable. The distribution according to population characteristics is shown in Table 1. The median age was 52 years (ranging from 13 to 60 years), including 36 patients (12 %) younger than 46 years. The ratio of male to female patients was 1.03. The distribution of the morphologic types of AML according to the FAB classification is shown in Table 1.

**Table 1:** Characteristics of study population (603 patients) and of patients who were randomized, by treatment arm

Characteristic	Total population, *N* (%)	Randomized	
		Intensive therapy, *N* (%)	Standard therapy, *N* (%)
Age (years)			
<46	72 (12)	18 (11)	18 (12)
46–55	363 (60)	93 (66)	104 (59)
56–60	168 (28)	47 (30)	35 (22)
Sex			
Male	307 (51)	81 (51)	78 (50)
Female			
FAB type			
M1	106 (18)	26 (17)	24 (15)
M2	187 (31)	48 (30)	43 (27)
M3	15 (2)	3 (2)	2 (1)
M4	140 (23)	46 (29)	43 (27)
M5	115 (19)	26 (17)	37 (24)
M6	33 (5)	9 (6)	8 (5)
M7	3 (1)	–	–
White cell count × 10^9^/L at diagnosis			
<25	363 (60)	97 (61)	94 (60)
25–100	168 (28)	46 (29)	45 (29)
≥100	72 (12)	15 (10)	18 (12)
Platelet count × 10^9^/L at diagnosis			
<50	282 (48)	75 (48)	80 (51)
≤50	321 (53)	83 (53)	77 (49)
Cytogenetic group^a^			
Good	18 (3)	9 (6)	7 (4)
Intermediate	92 (15)	28 (18)	26 (17)
Poor	46 (8)	13 (8)	9 (6)
Inconclusive	447 (74)	108 (68)	115 (73)
No. of courses needed to reach CR			
1	297 (81)	134 (85)	122 (78)
>1	70 (19)	24 (15)	35 (22)
Total	603 (100)	158 (100)	157 (100)

^a^Good prognosis = t(8;21), t(15;17), inv16. Intermediate prognosis = normal metaphases and –Y. Poor prognosis = trisomy 8, 5q-, monosomy 5 and 7 and all other cytogenetic abnormalities, including complex abnormalities

CR was achieved in 367 patients (61 %), and most remissions (81 %) were reached after one course of induction therapy. In addition, 157 patients did not achieve CR (26 %) and 79 patients died before evaluation of response (13 %). Out of 367 CR patients, 52 have not been randomized (for details, see Fig. 1). Finally, 315 patients were randomly assigned to two groups: 158 patients for the short intensive consolidation therapy and 157 for the standard consolidation and maintenance therapy. Table 1 shows the patient characteristics in both randomized groups. The patient and disease characteristics (i.e. age, sex, FAB classification, WBC and platelets on admission, cytogenetics) which have been reported to be of prognostic value were similar in the two treatment arms, except for the number of courses needed to reach CR, which favoured the intensive consolidation group [3]. In the intensive group, 149 (94 %) patients started the allocated treatment, 3 received standard consolidation/maintenance, 1 received another treatment and 5 received no treatment at all, whereas in the standard group 151 (96 %) patients started the allocated treatment, 3 received the intensive treatment and 4 received no treatment. The number of patients who completed their assigned treatment were 89 (56 %) in the intensive consolidation group and 104 (66 %) in the standard consolidation and maintenance group. Reasons for exclusion or not completing the assigned treatment in the two randomized arms are outlined in Table 2. Early relapse occurred more frequently in the standard group, whereas toxicity and refusal were more frequent in the intensive group. All patients were maintained in their assigned group for the analysis of the results to comply with the intention-to-treat principle.

**Table 2:** Reasons for going off-protocol treatment at each step according to the randomized treatment arm

Category	Intensive consolidation group (*N* = 158), *n* (%)	Standard consolidation and maintenance group (*N* = 157), *n* (%)
Normal completion of assigned treatment	89 (56)	104 (66)
Reasons for going off-protocol treatment		
Early relapse	6 (4)	31 (20)
Toxic effect	48 (31)	10 (6)
Refusal to undergo treatment	10 (6)	3 (2)
Protocol violation	3 (2)	7 (4)
Loss to follow-up or other reason	2 (1)	2 (1)

Table 3 shows the rates of relapse and of death in CR as a first event, along with the corresponding causes of death, according to both treatment groups. When comparing the intensive with standard group, the relapse rate was higher in the standard group, whereas the death rate in CR in the intensive group exceeded the rate of the standard group. The main cause of death in CR was infection. Grades 3 and 4 toxicities occurred more frequently in the intensive consolidation arm despite the shorter treatment period of this arm (Table 4). Especially toxicities related to the more profound cytopenias, such as infections and haemorrhages, were increased in the intensive consolidation arm.

**Table 3:** Type of first events (relapse or death in first CR) according to the randomized treatment arms

Category	Randomized intensive therapy (*N* = 158), *n* (%)	Randomized standard therapy (*N* = 157), *n* (%)
Relapse of leukaemia	91 (58)	121 (77)
Death during first CR	34 (22)	5 (3)
Causes of death		
Infection	16 (10)	3 (2)
Bleeding complications	11 (7)	–
Other	7 (4)	2 (1)
Alive in first CR	33 (21)	31 (20)

**Table 4:** Grade 3 and grade 4 toxicities observed during therapy in both treatment arms

Category grade 3/4	Intensive therapy (*N* = 158), *n* (%)	Standard therapy (*N* = 157), *n* (%)
Haemorrhage	17 (11)	3 (2)
Infections	43 (28)	12 (8)
Liver	12 (8)	10 (7)
Nausea, vomiting	22 (15)	10 (7)
Diarrhoea	6 (4)	1 (1)
Renal	3 (2)	0 (0)
Cardiac	8 (5)	1 (1)
Neurotoxic	4 (3)	1 (1)

**Fig. 2:**
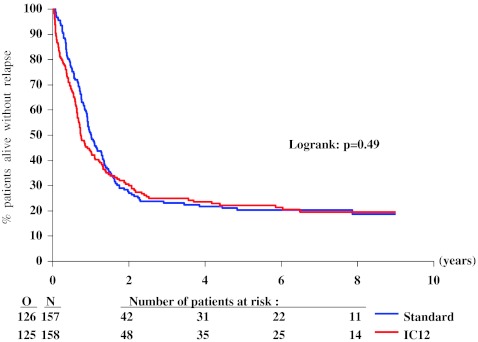
Kaplan–Meier plot of relapse-free survival according to the randomized arm. *N* number of patients, *O* observed number of events (relapse or death in first complete remission), *Standard* standard consolidation and maintenance therapy, *IC12* intensive consolidation therapy

**Fig. 3:**
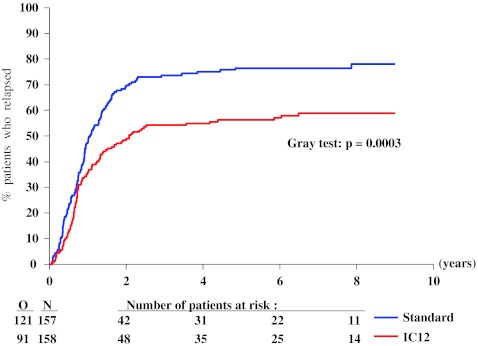
Cumulative incidence of relapse according to randomized arm. *N* number of patients; *O* observed number of events (relapse), considering death in first complete remission as a competing risk; *Standard* standard consolidation and maintenance therapy; *IC12* intensive consolidation therapy

**Fig. 4:**
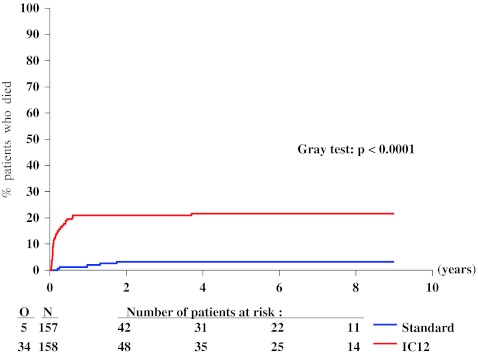
Cumulative incidence of death in first complete remission according to randomized arm. *N* number of patients; *O* observed number of events (death in first complete remission), considering relapse as a competing risk; *Standard* standard consolidation and maintenance therapy; *IC12* intensive consolidation therapy

**Fig. 5:**
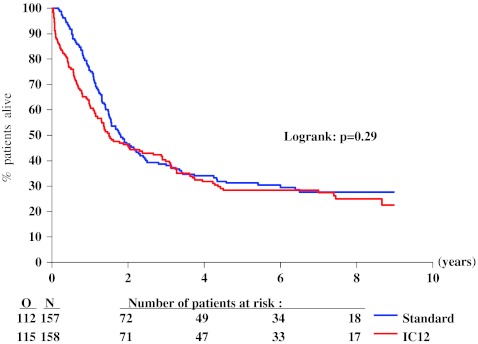
Kaplan–Meier plot of survival according to the randomized arm. *N* number of patients, *O* observed number of deaths, *Standard* standard consolidation and maintenance therapy, *IC12* intensive consolidation therapy

The median length of follow-up of surviving patients was 7.5 years. At 4 years after randomization, the probability of RFS in the intensive group was 24 % (SE 3 %) and 22 % (SE 3 %) in the standard group. Figure 2 shows the Kaplan–Meier curves of the two groups. There was no significant difference among the two groups (*P* = 0.49 by the log-rank test): HR, 1.09 (95 % CI 0.85 to 1.40). Figure 3 shows a lower 4-year relapse incidence rate in the intensive group than in the standard group: 55 % (SE 4 %) versus 75 % (SE 4 %; *P* = 0.0003). Figure 4 indicates that the 4-year incidence of death in first CR was 22 % (SE 3 %) in the intensive group and 3 % (SE 1 %) in the standard group (*P* < 0.0001). The treatment HR regarding the risk of relapse was 0.83 (95 % CI 0.63 to 1.09), and regarding the risk of death in CR was 7.3 (95 % CI 2.9 to 18.8). At 4 years, the probability of survival in the intensive group was 32 % (SE 4 %) and 34 % (SE 4 %) in the standard group. There was no significant difference among the two groups (Fig. 5, *P* = 0.29 by the log-rank test; HR 1.15, 95 % CI 0.89 to 1.49). After exclusion of the 36 patients younger than 46 years, in the remaining 279 patients aged 46 to 60 years, the OS difference between both groups (intensive versus standard consolidation) remained unchanged: HR 1.07, 95 % CI 0.75 to 1.54; *P* = 0.61. Analyses adjusted for the slight treatment imbalance regarding the number of courses to reach CR provided very similar results (data not shown). The median survival after relapse was 6 months in both arms with 12 % alive at 4 years after relapse in the standard arm and 11 % in the intensive consolidation arm (HR 0.99; 95 % CI 0.74 to 1.32).

Subsequent analyses for exploratory purposes with subgroups were performed. The comparison of overall survival of a high-risk group, consisting of randomized patients older than 55 years, who needed more than one course of induction treatment to reach CR and who did have a high WBC count (>100 × 10^9^/L) with the standard risk group (other randomized patients). In the latter group (173 patients), the intensive arm resulted in a 34 % 4-year survival rate compared to 43 % in the standard consolidation arm (HR, 1.32; 95 % CI 0.92 to 1.90; *P* = 0.13), while in the high-risk group (142 patients) the outcome was reverse: 4-year survival rate of 30 % in intensive arm versus 23 % in the standard arm (HR, 0.96; 95 % CI 0.66 to 1.40; *P* = 0.83).

We did not evaluate cytogenetic characteristics separately because of the insufficient number of patients with known cytogenetic profiles. Comparisons according to whether patients were treated in EORTC centres or in GIMEMA centres have been performed as well. Patients (*N* = 65) treated in the EORTC centres showed a trend in favour of intensive versus standard arm either in terms of RFS (HR, 0.88) and OS (HR, 0.78). In GIMEMA centres the reduction in relapse incidence could not be compensated by the increase in TRM observed in the intensive versus standard arm, resulting in an HR of 1.14 regarding RFS and an HR 1.25 regarding OS. The standard consolidation and maintenance arm differed between the two cooperative groups leading to a total dose of 7.4 g cytarabine in the EORTC centres versus 3.6 g in the GIMEMA centres and a dose of 315 mg and 240 mg daunorubicin, respectively, while the Italian patients received additional thioguanine. The 4-year OS rate of 124 Italian patients and the 33 EORTC patients was completely identical: 34 % in both groups. We also performed analyses to test whether a learning effect in time existed. Dividing the group of randomized patients in two equal cohorts according to the period of randomization, the treatment comparison regarding RFS and OS remained unchanged (data not shown).

## Discussion

This study did not show a survival advantage of treatment with two intensive consolidation courses containing high-dose cytarabine, when compared with a regimen consisting of standard consolidation and six maintenance courses containing standard dose cytarabine. The Italian patients in this study received a similar version of standard maintenance treatment. The 4-year survival of the 33 patients in the EORTC and the 124 patients in the GIMEMA centres treated with standard consolidation and maintenance therapy was 34 % (SE 8 %) and 34 % (SE 4 %), respectively. The toxicity patterns of both standard consolidation/maintenance regimens were very similar as well (data not shown). Therefore, all patients were analysed in the assigned group. The 4-year leukaemia relapse incidence was significantly (*P* = 0.0003) lower in the intensified regimen group (55 %) compared to the less intensive standard group (75 %), confirming the higher anti-leukemic activity of the intensive consolidation courses. Nevertheless, the higher toxicity of the intensified treatment led to a significantly higher mortality incidence in first CR (22 % versus 3 %; *P* < 0.0001). Because of this high toxicity, 31 % of patients in the intensive group did not finish the complete schedule, in contrast to only 6 % of patients in the group treated with standard post-remission chemotherapy. In the parallel study of the EORTC and GIMEMA Cooperative Leukemia Groups in a younger age group of 576 patients with a median age 33 years, the dropout due to toxicity after IC1 was only 18 % compared to 27 % (42 patients) after IC1 in patients older than 45 years in this study [22]. This indicates that administration of intensive consolidation courses is more problematic in patients older than 45 years. A limited number of studies addressed the impact of dose escalation of cytarabine as post-remission treatment in AML in this specific age group. Cassileth et al. showed that the combination of amsacrine and high-dose cytarabine as a single-course post-remission treatment, resulted in a higher event-free survival and better overall survival (only significant in patients younger than 41 years) than standard maintenance therapy, but this regimen exhibited high toxicity, especially in older patients [16]. Fopp et al. showed that one cycle of high-dose cytarabine in post-remission treatment, when compared to one cycle of standard dose cytarabine, exhibited a significantly higher RFS in patients older than 40 years and reduced the hazard of relapse [20]. This study also showed a trend for improved survival using one cycle of high-dose cytarabine. Both arms included daunomycin in the consolidation courses [20]. Because only one cycle of post-remission treatment was shown to be effective, more patients could tolerate the adverse effects of treatment [17, 20]. In contrast, Weick et al. observed that consolidation with high-dose cytarabine increased toxicity, but this approach did not improve OS or RFS compared to consolidation with standard dose cytarabine [19]. In this Southwest Oncology Group study, cytarabine was combined with daunorubicin in all schedules. Bradstock et al. evaluated the difference between high-dose cytarabine versus low or standard dose cytarabine as a consolidation treatment, after CR induction containing high-dose cytarabine, and they showed no difference for survival, relapse-free survival at 3 years or cumulative incidence of relapse. The high-dose cytarabine arm consisted of one course only and the standard cytarabine arm of two courses only, fewer courses than administered in most protocols. Cytarabine was combined with idarubicin and etoposide in all courses [8]. This study did not demonstrate a benefit of the intensive consolidation arm in any of the three cytogenetic groups [8]. Büchner et al. compared after three intensive courses of remission induction and consolidation therapy the efficacy of 3-year maintenance versus only one additional intensive consolidation course containing high-dose Ara-C. Patients treated with 3-year maintenance seem to benefit in terms of a better RFS, but the OS was not different in both groups [28]. In our study we could not use cytogenetic nor molecular characteristics as discriminating prognostic factor between short intensive and prolonged nonintensive post-remission treatment which might lead to certain imbalances. A few studies indicated that non-poor risk cytogenetic characteristics may favour intensive consolidation [18, 28], similarly to the more favourable response after adding anti-CD33 immunotoxin conjugate gentuzimab–ozogamicin to remission or consolidation courses in the favourable or intermediate risk cytogenetic groups [29].

Currently, several alternative treatment modalities may be considered for the patient population older than 45 years of age. Recently, escalation of the dose of daunorubicin to twice the conventional dose in the remission induction schedule of AML patients older than 60 years resulted in an increased overall survival in the age cohort between 60 and 65 years [30, 31]. Incorporation of this escalated dose of daunorubicin in the post-remission therapy is a challenging option, but increased cardiotoxicity may result from this dose intensification [30]. GO has been used as single agent for post-remission therapy in AML patients older than 60 years. GO post-remission therapy consisted of three courses GO (6 mg/m^2^) every 4 weeks. Only 65 of the 113 patients completed the three courses. The 5-year RFS was 17 % in the GO arm versus 16 % in the 119 patients who did not receive any post-remission therapy [32].

Autologous peripheral stem cell transplantation might be an interesting treatment modality [33]. Using this approach, the haematological recovery is quite fast, while this treatment exhibits the outcome advantages of transplantation. In the AML 10 study of the EORTC and GIMEMA LCG [34], one of the three arms used daunorubicin as anthracycline, but the patients without a donor received an autologous stem cell transplantation after the first consolidation course which was identical to the intensive consolidation course of the AML 8B study. The 4-year survival rate of the 148 patients in the same age group (46 to 60 years) without an HLA identical sibling and randomized for daunorubicin was 36 % (SE 4.0 %), similar to the 4-year survival rate, and 32 % (SE 4 %) observed in the intensive arm of this study. This indicates that replacement of the second consolidation course by autologous stem cell transplantation has no major impact on long-term survival as observed in the randomized AML 8a study performed at the same time in a younger age cohort [22]. Another promising treatment modality nowadays is reduced intensity conditioning allogeneic stem cell transplantation, which has the aim to exhibit the well-documented immune graft versus tumour effect, while attempting to control or to overcome toxicity. Promising results have been shown in patients with AML older than 50 years [35]. Prospective randomized studies are needed to verify this effect.

In conclusion this study showed that the lower relapse rate when using intensified post-remission treatment in patients with AML aged between 46 and 60 years was counterbalanced by higher toxicity. Future prospective studies should focus on the use of high-dose therapy consisting of a limited number of courses with attention to optimal supportive care and differences in outcome according to prognostic factors, especially cytogenetic profiles, as a result of which possibly intensive treatment could become the treatment of choice. Alternative treatment modalities as post-remission treatment, especially autologous peripheral blood stem cell transplantation and reduced intensity conditioning allogeneic stem cell transplantation, should be tested in the framework of prospective randomized trials. Moreover various prognostic factors, especially cytogenetic and molecular characteristics, should identify patients who may benefit of the treatment intensified by increased dosages of cytarabine and anthracyclines.

## Appendix

The following centres and investigators from the EORTC Leukaemia Cooperative Group participated in this study: Austria—Innsbruck, University Klinik, (J. Thaler); Belgium—Brugge, Hospital St. Jan (D. Selleslag); Brussel Hospital Bordet-Erasme(W. Ferremans); France—Nice, Centre Antoine Lacassagne (A Thyss); Paris, Hospital Hotel-Dieu (J-P Marie, A. Vekhoff); Villejuif, Institut Gustave Roussy (M. Hayat); The Netherlands—Enschede, Medisch Spectrum Twente (M. Schaafsma); Hertogenbosch, Jeroen Bosch Ziekenhuis (J. Burghouts); Nijmegen, Radboud University Medical Centre (T. de Witte, P. Muus); Amsterdam, Onze Lieve Vrouw Gasthuis (B. De Valk); Portugal—Coimbra, Institute of Oncology (G. Teixeira); Porto, San Joao (M. Ribeiro); and Turkey—Ankara, Ibnui Sina Hospital (M.Beksac), Germany: Heidelberg, Kopfklinik,(A. Ho?), Croatia, Zagreb, Hopital Rebro (B. Labar), Hospital Merkur (B. Jaksic). The following centres and investigators from the GIMEMA group participated in this study: Italy—Reggio Calabria, Dipartimento Emato-Oncologia A.O. “Bianchi-Melacrino-Morelli” (A. Neri); Roma, U.O.C. Ematologia—Ospedale S.Eugenio (G. Papa); Roma, Università degli Studi “Sapienza”—Dip Biotecnologie Cellulari ed Ematologia—Divisione di Ematologia (F. Mandelli); Roma, Università Cattolica del Sacro Cuore—Policlinico A. Gemelli (B. Bizzi); Roma, Divisione di Ematologia—Ospedale S. Camillo (A. De Laurenzi); Palermo, Azienda Ospedaliera—Policlinico Divisione di Ematologia (P. Citarrella); Palermo, Div. di Ematologia—A.O. “V. Cervello” (F. Caronia); Palermo, Divisione di Ematologia con trapianto di midollo—Università degli Studi di Palermo—A.U. Policlinico ( A. Cajozzo); Parma, Cattedra di Ematologia CTMO Università degli Studi di Parma (V. Rizzoli); Pavia, Div. di Ematologia IRCCS Policlinico S. Matteo (c. Bernasconi); Nuoro, Sez. di Ematologia Clinica Ospedale San Francesco (A. Gabbas); Ancona, Clinica di Ematologia, Osp. Riuniti di Ancona (P. Leoni); Torino, Div. di Ematologia “Molinette” Osp. Maggiore S. G. Battista (L. Resegotti, A. Pileri); Perugia, Sezione Ematologia ed Immunologia Clinica—Ospedale S.Maria della Misericordia (F. Grignani); Aviano, Centro di Riferimento Oncologico (S. Monfardini); S.G. Rotondo, Istituto di Ematologia—IRCCS Ospedale Casa Sollievo della Sofferenza (M. Carotenuto); Latina, Divisione di Ematologia Ospedale “Santa Maria Goretti” (L. Deriu); Pesaro, Div. di Ematologia di Muraglia—CTMO Ospedale San Salvatore (G. Lucarelli); Bari, Unità Operativa Ematologia 1—Università degli Studi di Bari—Padiglione Chini—3°piano (V. Liso), Catanzaro, Azienda Ospedaliera Pugliese Ciaccio—Presidio Ospedaliero A. Pugliese—Unità Operativa di Ematologia (A. Alberti); Avellino, Az.Ospedaliera S.G.Moscati (E.Volpe); Milano, Ospedale Niguarda “Ca Granda” (F. De Cataldo); Napoli, A.S.L. Napoli 1 Ospedale San Giovanni Bosco (R. De Biasi); Napoli, Azienda Ospedaliera Universitaria-Università degli Studi di Napoli “Federico II”—Facoltà di Medicina e Chirurgia (B. Rotoli); Napoli, Azienda Ospedaliera di Rilievo Nazionale “A. Cardarelli” (R. Cimino); Cagliari, ASL N.8—Ospedale “A. Businco”—Unità Operativa di Ematologia e Trapianto di Midollo (G. Broccia); Pescara, U.O. Ematologia Clinica—Azienda USL di Pescara (G. Torlontano); Potenza, Ematologia—Ospedale San Carlo (F. Ricciuti); Sassari, Serv. di Ematologia Ist. di Ematologia ed Endocrinologia (M. Longinotti); Cremona, Sezione di Ematologia C.T.M.O. Istituti Ospitalieri, (E. Bianchini). Cytology committee: M. Cadiou, M. Bernier, G. den Ottolander, U. Jehn, W. Sizoo, G. L. Castoldi, S. Fenu, and V. Liso. Cytogenetic committee: A. Hagemeijer, G. Alimena, A Bernheim, and A. Zaccaria. Study coordinator: R. Zittoun. Statistician: S. Suciu. Data managers: G. Solbu and M. Dardenne.
